# Effects of Feeding Methionine Hydroxyl Analogue Chelated Zinc, Copper, and Manganese on Growth Performance, Nutrient Digestibility, Mineral Excretion and Welfare Conditions of Broiler Chickens: Part 1: Performance Aspects

**DOI:** 10.3390/ani15030421

**Published:** 2025-02-03

**Authors:** Hoang Duy Nguyen, Amy Fay Moss, Frances Yan, Hugo Romero-Sanchez, Thi Hiep Dao

**Affiliations:** 1 School of Environmental and Rural Science, Faculty of Science, Agriculture, Business and Law, University of New England, Armidale, NSW 2351, Australia; hnguye66@myune.edu.au (H.D.N.); amoss22@une.edu.au (A.F.M.); 2 Novus International, Inc., 20 Research Park Drive, St. Charles, MO 63304, USA; frances.yan@novusint.com (F.Y.); hugo.romero@novusint.com (H.R.-S.); 3 Faculty of Animal Science, Vietnam National University of Agriculture, Trau Quy Town, Gia Lam District, Hanoi 100000, Vietnam

**Keywords:** chelated trace minerals, meat chicken, performance, carcass, digestibility

## Abstract

This floor pen study explored the effects of feeding mineral methionine hydroxyl analogue chelates (MMHACs) zinc (Zn), copper (Cu), and manganese (Mn) on growth performance, carcass processing weight and quality, nutrient digestibility, gizzard erosion score, and bone parameters of broilers. One-day-old Ross 308 male chicks (*n* = 384) were randomly allocated to four dietary treatments with eight replicate pens per treatment and 12 birds per pen. The treatments were as follows: (1) inorganic trace mineral ZnSO_4_ 110 ppm, CuSO_4_ 16 ppm, MnO 120 ppm (ITM); (2) MMHAC Zn 40 ppm, Cu 10 ppm, Mn 40 ppm (M10); (3) Inorganic trace mineral ZnSO_4_ 110 ppm, tribasic copper chloride 125 ppm, MnO 120 ppm (T125); (4) MMHAC Zn 40 ppm, Cu 30 ppm, Mn 40 ppm (M30). The birds were fed nutritionally complete wheat sorghum soybean meal-based diets from days 0 to 42 with three feeding phases including starter (days 0–10), grower (days 10–21), and finisher (days 21–42). The findings revealed that birds on the T125, M10, and M30 groups possibly had higher feed intake (*p* = 0.052) and higher weight gain (*p* < 0.063) than birds on the ITM group from days 0 to 42. Furthermore, birds fed the M30 diet had higher thigh and drumstick weights compared to those fed the ITM diet at day 42 (*p* = 0.05). Additionally, birds offered the M30 diet had increased ileal Cu digestibility compared to birds offered the M10 and ITM diets at day 21 (*p* = 0.006). Gizzard erosion scores and bone parameters were similar between the dietary treatments. Hence, the supplementation of MMHACs to broiler diets at 30 ppm may be a more suitable strategy to increase weight gain and the thigh and drumstick processing weight while maintaining the bone health of broiler chickens.

## Simple Summary

A high intake of inorganic minerals in conventional broiler diets is wasteful and directly impacts the environment since most inorganic minerals are excreted as waste and the body stores them to a very small extent. Chelated trace minerals, on the other hand, are more available for digestibility and absorption resulting in less mineral release into the environment following its utilisation in broiler diets. This study determined the effects of supplementing chelated trace minerals on broiler chickens by comparing growth performance, carcass processing weight and nutrient digestibility in broilers fed conventional inorganic trace mineral sources and those fed diets with lower trace mineral levels and with added chelated minerals. The results of this study may help to improve the economic and environmental sustainability of the broiler industry.

## 1. Introduction

Trace minerals play a vital role in the production of broilers and are essential for the proper functioning of various metabolic processes [1]. Trace minerals are also part of metal coenzymes involve in complex intermediary metabolism, immune function, and hormone secretion processes [2]. Due to the concerns that inadequate dietary mineral levels and mineral interactions may cause a deficiency of trace minerals in chickens, it is common practice in the poultry industry to supplement diets with inorganic trace minerals with a large safety margin. However, Lee et al. [3] indicated that a high intake of inorganic minerals is wasteful and harmful to the environment since most inorganic minerals are excreted as waste, and the body stores them only to a very small extent. Indeed, there is alarm surrounding the mineral accumulation in the environment and the associated economic losses, especially for zinc (Zn) and copper (Cu). This has raised concerns about the practice of mineral over-supply in broiler diets and prompted the search for mineral sources, which can be used at lower levels but still ensure birds’ nutritional requirement, growth performance, and meat quality [4]. Furthermore, it appears that high dietary mineral levels increase mineral interactions that may consequently reduce mineral bioavailability and absorption in the intestine [5]. This problem may be solved by replacing inorganic trace minerals in broiler diets with organic trace minerals such as chelated trace minerals as organic trace minerals have been demonstrated to have higher bioavailability than their inorganic counterparts [6]. Brooks et al. [7] suggested that the higher bioavailability of organic trace minerals compared to the inorganic trace mineral sources may be attributed to their effects on diminishing the interactions of trace minerals with phytate, fibre, and macro minerals such as calcium and/or phosphorus; thereby, organic trace minerals undergo fewer antagonistic reactions with these dietary components during digestion. Therefore, organic trace minerals can be used in broiler diets at lower levels compared to the inorganic trace minerals without negative impacts on growth performance while reducing mineral excretion to the environment [8]. This is of relevance as the accumulation of trace minerals, particularly Zn, Cu, and manganese (Mn), in the environment contributes to the environmental burden.

While the benefits of organic trace minerals are widely recognised, various organic mineral sources can be used in poultry production. The Association of American Feed Control Officials gave explanations for a range of organic trace mineral complexes that are commonly used in the livestock sector [9]. These complexes consist of mineral amino acid chelate, mineral proteinate, mineral polysaccharide complex, and mineral amino acid complex [8]. Among these sources, the benefits of supplementing chelated trace minerals in broiler diets have been demonstrated [10,11]. For instance, feeding a blend of 50% inorganic Zn and Mn and 8 ppm Cu from sulphates and 50% organic chelated Zn, Mn, and Cu has been shown to improve growth performance and carcass yield in broiler chickens [10]. Furthermore, other investigators have reported that birds offered chelated trace minerals at 100% of the inorganic trace mineral levels in the mineral premix had the highest feed efficiency, performance index, tibia ash, Zn, Cu, and Mn levels, and serum total antioxidant capacity and the lowest malondialdehyde level compared to those offered diets with 100% inorganic trace minerals or lower inclusion rates of chelated trace minerals [11,12]. Feeding broilers with high levels of dietary inorganic Cu (e.g., 125 ppm) as a growth promotor is a common industry practice in some world areas due to the cost-effectiveness and convenience; however, optimal Cu levels in broiler diets remain controversial [13]. It has been reported that dietary Cu supplementation above the bird’s requirement increased growth performance and nutrient digestibility in broiler chickens [14,15]. These beneficial effects of dietary Cu supplementation are most likely associated with Cu antimicrobial properties as an increase in the numbers of pathogenic bacteria populations has been shown to decrease body weight (BW) gain in birds [16,17,18]. Additionally, Cu supplementation at high levels may help to control microbes in feed, increase activities of intestinal lipase and other enzymes, and stimulate the secretion of growth hormone [16,19,20]. However, high dietary Cu supplementation may also present certain disadvantages. For example, the excessive supplementation of inorganic Cu, particularly Cu sulphate (e.g., 150 ppm or higher), has been shown to reduce growth performance in birds [21,22,23]. It is possible that the biologically toxic nature of inorganic Cu at high levels damages the liver and kidneys leading to decreased growth performance [24,25]. Furthermore, as inorganic Cu is usually digested to a very small extent, unabsorbed Cu would be excreted in bird excreta, causing environmental pollution and reducing water quality and soil fertility [13,26,27,28]. High dietary levels of inorganic Cu may also cause gizzard erosion resulting in a decrease in the liveability rate, growth performance, and health conditions of broilers [29,30]. The dietary supplementation of organic instead of inorganic Cu sources at appropriate levels showed growth-promoting effects in piglets [31,32] while minimising the aforementioned negative impacts of Cu supplementation on the environment. However, reports comparing the effects of Cu as a growth promoter using inorganic Cu salts versus a reduced rate of organic Cu sources are scarce in the literature on poultry [33]. Given the increasing Cu price in the market [13] and possible effects of high dietary Cu supplementation, it is necessary to reassess the optimal Cu level and source in broiler diets to promote industry economic and environmental sustainability. Thus, the main objective of the current study was to determine the growth performance, carcass processing weight and quality, nutrient digestibility, gizzard erosion score, and bone parameters of broilers offering mineral methionine hydroxyl analogue chelates (MMHACs) Zn, Cu, and Mn at recommended rates (40, 10, and 40 ppm of Zn, Cu, and Mn, respectively) in a traditional diet. Additionally, further treatments investigated the effects of Cu as a growth promoter under the same conditions using either an inorganic Cu salt (125 ppm) or a reduced rate of Cu chelate (30 ppm). It is hypothesised that the supplementation of MMHACs at reduced rates can replace high inorganic Cu salt in broiler diets without affecting the growth performance, carcass traits, and bone quality of broilers. The effects of the dietary treatments on the excreta nitrogen and mineral levels, environmental conditions, and welfare status of broilers were also determined. The findings will be reported in the subsequent companion paper (part 2) to maintain the focus and proper length of each paper.

## 2. Materials and Methods

The study was conducted at the Centre of Animal Research and Teaching at the University of New England (UNE), Armidale, New South Wales, Australia. The study was approved by the UNE Animal Ethics Committee (Approval number: ARA23-004) and fulfilled the criteria for the use and care of animals for scientific purposes as outlined in the Australian Code of Practice [34].

### 2.1. Experimental Design and Diets

The experimental broiler shed was fully enclosed and tunnel-ventilated with temperature and humidity control. During the brooding period (days 0–10), the indoor temperature was regulated using 3 electric brooders equipped with thermostatic controls. A total of 32 pens were used. A manual feeder and two nipple drinkers were arranged for each pen. The area within each pen, excluding the space occupied by the feeder and drinkers, was approximately 1 m^2^. Day-old Ross 308 males (*n* = 384) were randomly distributed to 4 dietary treatments with 8 replicate pens comprising 12 chicks per pen per treatment. The initial pen weights were similar among the treatments (*p* > 0.05). Birds were raised in floor pens containing hardwood shavings as the bedding material in an environmentally controlled room over 42 days of the feeding experiment. All chicks received feed and water within 12 h of hatch. Birds were vaccinated with the infectious bronchitis virus (IBV) and Newcastle disease virus (NDV) combination vaccine (1 × IBV/NDV vaccine) at hatch as per the vaccination programme of the hatchery (Aviagen Chicken Hatchery, Goulburn, New South Wales, Australia). Birds had ad libitum access to the feed and water over the entire study. The lighting, temperature, and ventilation conditions at the broiler shed followed Ross 308 recommendations [35]. Specifically, a ‘23 h on–1 h off’ lighting regime was applied for the first 3 days, followed by ‘20 h on–4 h off’ to 7 days and finally, ‘18 h on–6 h off’ for the remainder of the study. An initial room temperature of 32 ± 1 °C was maintained for the first week, which was gradually decreased to 21 ± 1 °C by the end of the third week, and maintained at this temperature until the end of the study. Birds were checked twice daily to assess their overall health conditions and to ensure feed and water were always present. The feed was pelleted at 65 °C and was provided in crumble form during the starter phase (days 0–10), transitioning to pellets during the grower phase (days 10–21) and the finisher phase (days 21–42). The treatments were as follows: (1) inorganic trace mineral ZnSO_4_ 110 ppm, CuSO_4_ 16 ppm, MnO 120 ppm (ITM); (2) MMHAC Zn 40 ppm, Cu 10 ppm, Mn 40 ppm (M10); (3) Inorganic trace mineral ZnSO_4_ 110 ppm, tribasic copper chloride 125 ppm, MnO 120 ppm (T125); and (4) MMHAC Zn 40 ppm, Cu 30 ppm, Mn 40 ppm (M30, Table 1). The levels of Zn, Cu, and Mn in the ITM treatment followed the Ross 308 nutritional recommendations [36], while the levels of Zn, Cu, and Mn in the M10, M30, and T125 treatments were selected based on previously published reports [12,22,33,37,38,39,40]. In more detail, it has been shown that the supplementation of inorganic Cu salts at 125 ppm is less toxic while improving growth performance in broilers compared to the higher doses [22,38]. Additionally, a review conducted by Jensen [39] concluded that the dietary supplementation of inorganic Cu at concentrations above 125 ppm strongly increases gizzard erosion in broilers. However, feeding broilers with organic Zn, Cu, and Mn at 40-10-50 ppm, 40-30-40 ppm, 40-25-40 ppm, and 50-10-60 ppm has been demonstrated to increase the BW, BW gain (BWG), feed efficiency, and/or meat quality of broilers compared to the inorganic Zn, Cu, and Mn sources [37,40]. The MMHAC Zn, Cu, and Mn used in the study was MINTREX^®^ Zn, Cu, and Mn trace minerals from Novus International, Inc. (Chesterfield, MO, USA) with Zn, Cu, and Mn bound by methionine hydroxyl analogues in a 2:1 chelated molecule. The levels of supplemental methionine were reduced in the M10 and M30 diets to generate the same methionine levels across the treatments [41]. The vitamin–mineral premix with desirable Zn, Cu, and Mn levels and sources for each treatment were produced and provided by an experienced manufacturer (Rabar Pty Ltd., Beaudesert, QLD, Australia). Wheat was used as the filler in the diets.

Titanium dioxide was added to the grower diets at 0.5% as an inert dietary marker for digestibility measurement. The nutritional compositions of soybean meal, wheat, and sorghum were analysed through near-infrared reflectance spectroscopy (Foss NIR 6500, Hillerød, Denmark) and standardised using Adisseo calibration before diet formulation. Crude protein content of soybean meal, wheat, and sorghum were also measured by a nitrogen analyser (LECO Corporation, St. Joseph, MI, USA) with EDTA serving as a calibration standard prior to diet formulation. Diets were formulated using commercial feed formulation software (Concept 5, CFC Tech Services, Inc., Browerville, MN, USA). Nutrient levels in all diets met the nutritional requirement of broiler chickens according to Ross broiler nutrition specifications [36]. Gross energy, crude protein, dry matter, and mineral levels of mixed diets were determined using standard methods [42] to confirm formulated levels. Information regarding the diet composition and nutrient content are provided in Table 2 and Table 3.

### 2.2. Data Collection

#### 2.2.1. Growth Performance

Body weight as well as feed and water consumption were determined per pen by feeding phase. Then, the BWG and feed conversion ratio (calculated as feed to gain, FCR) were subsequently computed. Daily records of morbidity and mortality were maintained for subsequent calculations. To account for mortality, the FCR was adjusted by incorporating the BWG of dead birds into the live birds during each feeding phase. The feed intake (FI) was calculated by multiplying the BWG by adjusted-FCR. Each pen was equipped with a water barrel (maximum capacity of 28 L) which was calibrated based on the water volume and height of the barrel to ensure accurate measurements of water intake per pen. The water intake was calculated by subtracting the volume of water provided in the barrels within each feeding phase and the volume of remaining water left in the barrels at the end of the feeding phase. The water volumes in the barrels were re-measured if there were mortalities in the pens to ensure accurate calculations of water intake per bird. The overall liveability and European Productivity Index (EPI) were calculated for overall period as per the following equation.

$$EPI=(Liveability\times \frac{BW}{age})\times \frac{100}{FCR}$$

#### 2.2.2. Carcass Characteristics, Gizzard Erosion Score, and Bone Parameters

At day 42, 3 birds per pen were randomly selected, and then subjected to weighing, electrical stunning (MEFE CAT 44N, Mitchell Engineering Food Equipment, Clontarf, QLD, Australia), and euthanasia by cervical dislocation to collect samples. The number and severity of skin scratches and breast irritation/blisters, with breast muscle myopathies ‘woody breast’ and surface ‘white striping’ recorded. A scratch was noted when one or more linear signs of skin lesion were observed, and a breast blister was noted once it was equal to or larger than 0.5 cm^2^ following Allain et al. [43]. Skin scratch number and severity were scored as 0 for no scratches; 1 for small, superficial scratch or scratches; and 2 for scratch or wound (1.5 cm) following De Jong et al. [44]. Breast irritation was scored by inspecting the breast area following the scoring method described by De Jong et al. [44], where 0 indicated no irritation or redness of the breast; 1 indicated slight discoloration (redness) of the breast; 2 indicated large discoloration (redness) and the presence of small brown spots; and 3 indicated large brown spots or blister(s). The myopathies affecting the breast muscle, namely ’woody breast’ and surface ’white striping’, were also recorded. The woody breast scoring was conducted on whole breast fillets using the method outlined by Tijare et al. [45]. Specifically, fillets that were flexible throughout were designated a score of 0 (normal), while those exhibiting hardness mainly in the cranial region but flexibility elsewhere were assigned a score of 1 (mild); fillets that were hard throughout but showed flexibility in the mid- to caudal region were rated 2 (moderate); and fillets that were extremely hard and inflexible from the cranial to caudal tip received a score of 3 (severe). The scoring for breast white striping was conducted following the method described by Pedrão et al. [46], where scores were determined as follows: 0 = the absence of visible white stripes on the breast muscle; 1 = the presence of white striping, with fine stripes that might not cover the entire breast muscle; 2 = obvious white striping covering the breast muscle more thickly; and 3 = easily detectable white stripes with numerous thick bands. At day 42, the absolute and relative weights of the breast, drumstick, thigh, abdominal fat, and internal organs such as the liver, heart, and kidney were also assessed.

Gizzard samples collected from 3 birds per pen on day 42 were opened and assessed for gizzard erosion using the method described by Wijnen et al. [47]. Erosion was characterised by a dark discoloration of the koilin layer, as defined by Gjevre et al. [48]. The severity of gizzard erosions was graded on a scale: 0 for no erosions, 1 for a single erosion, 2 for 2 to 3 erosions, and 3 for more than 3 erosions.

Samples of the right tibia were obtained from 3 birds per pen on day 42 by completely removing the muscle tissue and subsequently weighing. These samples were then air-dried at 20 °C for 72 h, and the initial weights of the air-dried tibia samples were recorded. The samples were stored at 4 °C for further measurements including length, diameter, bone ash, and breaking strength. Tibia diameter (measured at the medial region of tibia) and length were determined by using a digital calliper, while the breaking strength of the tibias was assessed by using an Instron machine (LX 300 Instron Universal Testing Machine, Instron Corp., Norwood, MA, USA). Finally, the tibia samples were subjected to oven-drying at 105 °C during 24 h, followed by ashing at 600 °C during 13 h, to measure the oven-dried weight and ash content, respectively.

#### 2.2.3. Apparent Nutrient Digestibility

At day 21, 3 birds per pen were randomly selected, and then subjected to weighing, electrical stunning (MEFE CAT 44N, Mitchell Engineering Food Equipment, Clontarf, QLD, Australia), and euthanasia by cervical dislocation to collect ileal digesta samples. Ileal contents were collected by compressing gently the entire ileum (from Meckel’s diverticulum location to 1 cm before the ileocecal junction). The samples were added into 50 mL containers and preserved at −20 °C for further analysis. Samples of ileal digesta were freeze-dried by freeze dryer machine (Christ Alpha 1-4 LDplus, Osterode am Harz, Germany) until constant weights were achieved. Ileal digesta and feed samples were ground finely to particle sizes of 0.5 mm and then underwent duplicate titanium assay measurements using a colorimetric method as outlined by Short et al. [49]. If the difference between duplicates exceeded 5%, the run was repeated. The nitrogen content in both ileal digesta and feed samples was determined using a nitrogen analyser instrument (LECO Corporation, St. Joseph, MI, USA), calibrated with EDTA. The gross energy levels in ileal digesta and feed samples were measured using the Parr adiabatic oxygen bomb calorimeter machine (Parr Instrument Co., Moline, IL, USA), calibrated with benzoic acid as the standard. The mineral concentrations in both feed and ileal digesta samples were analysed by the inductively coupled plasma-optical emission spectrometry instrument (Agilent, VIC, Australia). Ileal digestibility coefficients for nitrogen (INDC), energy (IEDC), and minerals (IMDC) were calculated using the equations outlined by Jasek et al. [50].

(1) $$INDC=1-\left(\frac{{Ti}_{diet}\times N_{digesta}}{{Ti}_{digesta}\times N_{diet}}\right)$$

(2) $$IEDC=1-\left(\frac{{Ti}_{diet}\times {GE}_{digesta}}{{Ti}_{digesta}\times {GE}_{diet}}\right)$$

(3) $$IMDC=1-\left(\frac{{Ti}_{diet}\times M_{digesta}}{{Ti}_{digesta}\times M_{diet}}\right)$$

where the variables N_diet_ and N_digesta_ represented the nitrogen content in diet and ileal digesta samples, respectively. Similarly, GE_diet_ and GE_digesta_ were the gross energy levels in diet and ileal digesta, respectively, while M_diet_ and M_digesta_ referred to the mineral content in diet and ileal digesta, respectively. Additionally, Ti_diet_ and Ti_digesta_ were the titanium dioxide content in diet and ileal digesta samples, respectively.

### 2.3. Statistical Analysis

The obtained data were analysed using the R Commander (version 3.3.1, R Foundation for Statistical Computing, Vienna, Austria) to test statistical differences between the dietary treatments. The initial data were visually explored through boxplots, scatterplots, histograms, and normality testing using the Shapiro–Wilk test. Depending on whether the data distribution was approximately normal or non-normal, one-way ANOVA or non-parametric ANOVA (Kruskal–Wallis test) was employed to test statistical differences among the dietary treatments. Tukey’s post hoc test was used to separate the means following a significant ANOVA result. Data on liveability rates, woody breast score, breast white striping score, and gizzard erosion score were not normally distributed and thus were analysed by the Kruskal–Wallis test; however, no significant results were obtained for these variables. A *p*-value ≤ 0.05 was considered significant and a possible effect was recognised at 0.05 < *p*-value < 0.10.

## 3. Results

### 3.1. Growth Performance

The results on bird growth performance are shown in Table 4. The BWG, FI, FCR, and liveability rate were not different between the dietary treatments during starter phase from days 0 to 10. However, during the grower phase from days 10 to 21, birds fed the T125 diet had a lower FCR compared to birds fed the M10 diet (*p* = 0.021, Table 4). Additionally, birds fed the M10 and M30 possibly had a higher FI compared to those fed the T125 and ITM diets during the grower phase (*p* = 0.086, Table 4). During the finisher phase from days 21 to 42, birds offered the T125 diet possibly had a higher FI compared to birds offered the ITM diet (*p* = 0.057) and a lower liveability rate compared to birds offered the M10 and ITM diets (*p* = 0.085, Table 4). Over the entire study, birds offered the M10, M30 and T125 diets possibly had higher FI with the highest FI observed for T125 group (*p* = 0.052), and higher BWG with the highest BWG observed for the M30 group compared to those offered the ITM control diet (*p* = 0.063, Table 4). However, the EPI over the entire study and BW uniformity at day 42 were not different between the dietary treatments (Table 4).

The results regarding the water intake (WI) of the treatment groups are shown in Table 5. The dietary treatments did not affect WI during the starter phase. During the grower phase, birds fed the M10 diet possibly had higher relative WI per kg of BW compared to those fed the T125 diet (*p* = 0.057, Table 5). During the finisher phase, birds offered the M30 diet exhibited a higher absolute WI compared to birds offered the ITM diet (*p* = 0.020, Table 5). Over the entire study, higher values of absolute WI (*p* = 0.002), relative WI per kg of BW (*p* = 0.010), and water-to-FI ratio (*p* = 0.046) were observed in birds offered the M10 diet compared to those offered the T125 diet (Table 5).

### 3.2. Carcass Characteristics, Gizzard Erosion Score, and Bone Parameters

Carcass characteristics of the dietary treatments at day 42 are presented in Table 6.

**Figure 1:**
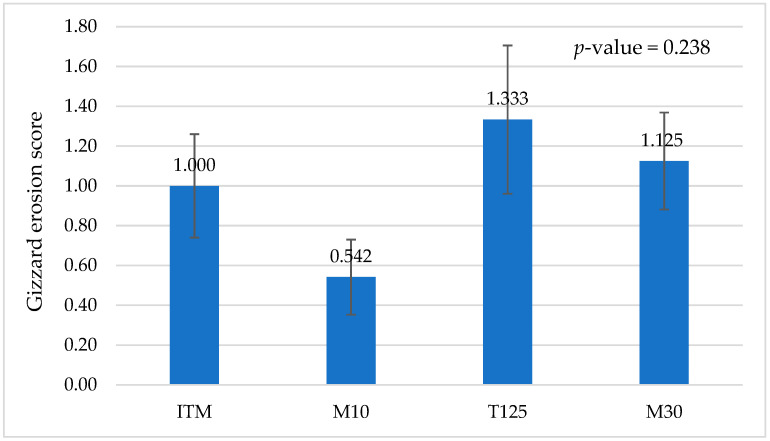
Gizzard erosion scores of broilers fed inorganic and methionine hydroxyl analogue chelated zinc, copper, and manganese at day 42. ITM: inorganic trace mineral ZnSO_4_ 110 ppm, CuSO_4_ 16 ppm, MnO 120 ppm; M10: mineral methionine hydroxyl analogue chelates Zn 40 ppm, Cu 10 ppm, Mn 40 ppm; T125: inorganic trace mineral ZnSO_4_ 110 ppm, tribasic copper chloride 125 ppm, MnO 120 ppm; M30: mineral methionine hydroxyl analogue chelates Zn 40 ppm, Cu 30 ppm, Mn 40 ppm. Vertical lines over the bars represent standard errors of the means.

Thigh and drumstick weights were higher in birds fed the M30 diet compared to birds fed the ITM control diet at day 42 (*p* = 0.050, Table 6). However, the weights of breast, fat pad, liver, heart, and kidney were not different between the dietary treatments. Breast blister and skin scratches were not observed on sampled birds at day 42. Similarly, gizzard erosion score was not different between the dietary treatments at day 42 (Figure 1). The percentage yield values (%) of all carcass parts, fat pad, liver, heart, and kidney were not significantly different between the dietary treatments (*p*-value > 0.05); thus, the data were not presented in this paper.

Tibia parameters including tibia weight, ash content, length, diameter, and breaking strength were not affected by the dietary treatments at day 42 (Table A1).

### 3.3. Nutrient Digestibility

The results on apparent mineral, nitrogen, and energy digestibility of the dietary treatments at day 21 are described in Table 7. Birds fed the M30 diet had higher Cu digestibility compared to those fed the M10 and ITM diets at day 21 (*p* = 0.006, Table 7). The digestibility of nitrogen, energy, and other minerals was not different between the dietary treatments at day 21 (Table 7). Negative digestibility values were observed for Zn and Mn in all treatments and Cu in M10 treatment at day 21, which may be due to the influence of secreted endogenous minerals in the digestive tract.

## 4. Discussion

It is widely accepted that organic minerals are more bioavailable as they undergo fewer antagonistic reactions with other dietary components during digestion compared to inorganic trace minerals [10]. Previous studies have demonstrated that higher trace mineral bioavailability might translate into improved tissue development, immune function, and growth performance in broilers [12,51,52]. Furthermore, chelated trace minerals are reported to have higher bioavailability compared to other inorganic and organic trace mineral sources [53,54,55]. These facts may partly explain the possibly higher BWG in birds fed the M10 and M30 diets compared to those fed the ITM diet in this study. The findings of this study align with the findings reported by Sirri et al. [12] that birds offered chelated Cu, Zn, and Mn had a higher BWG compared to those offered the inorganic mineral sources.

There was evidence that the dietary supplementation of Cu salts above the bird’s requirement improved the growth performance and nutrient digestibility in broiler chickens [14,15]. Thus, broiler diets with high Cu levels (e.g., 125 ppm) are widely used across the poultry industry for growth promotion in some world areas [13]. The mechanisms for the growth-promoting effects of high Cu levels in broilers are likely attributed to its antimicrobial actions, which are similar to antibiotics [16,18]. It is known that pathogenic organisms reduce BWG in birds [17]. The findings of this study suggest that higher dietary Cu levels either in inorganic (TBCC) or chelated forms (MMHAC) are more beneficial during the starter and grower phases. This might be due to Cu benefits in reducing gut pathogenic bacteria in young birds when their immune system may be not functioning well and their gut health is most challenged. However, during the finisher phase, higher dietary Cu levels particularly the inorganic form may not generate any beneficial effects and may even reduce the feed efficiency and liveability rate in birds. Thus, using the M30 diet in the starter and grower phases and the M10 diet during the finisher phase may be the best strategy to improve birds’ growth performance and health conditions. It has been reported that high dietary Cu levels stimulate the secretion of neuropeptide Y resulting in an increased FI [56,57]. This may partly explain the possibly higher FI in birds fed the T125 and M30 diets in the current study. Additionally, Wu et al. [58] reported that dietary Cu supplementation increased the BWG and FI but did not affect the FCR in broilers. Similar findings were observed in the current study. However, the reasons for the higher WI in birds fed the M10 diet compared to those offered the T125 diet in this study are unclear.

Previous studies have indicated that the supplementation of chelated trace minerals increased the carcass yield in broilers [10]. In the current study, birds fed the M30 diet had a higher processing weight of thigh and drumstick compared to those fed the ITM control diet at day 42. The higher carcass processing weight in birds fed chelated trace minerals such as MMHACs may be associated with higher mineral bioavailability and improved general health conditions resulting in increased nutrients being directed to muscle deposition rather than supporting immune function in the respective group [10]. Additionally, there is evidence that broilers fed high Cu diets such as 150 ppm Cu sulphate had higher weights of overall carcass, breast, and wing compared to those offered diets containing 8 ppm Cu sulphate [59]. This finding is supported by Philpot et al. [60], who reported that feeding Cu as TBCC at high levels (270 ppm in addition to 12 ppm Cu from mineral premix) during the finisher phase increased breast yield but did not affect wooden breast and white striping scoring in broilers. The increased carcass yield following Cu supplementation may be attributed to Cu effects on increasing concentrations of growth hormones and amino acid digestibility resulting in increased muscle synthesis [61,62,63,64]. Conversely, others observed no differences in carcass yield and weights of different carcass parts (breast, wing, thigh, and drumstick) in broilers fed diets containing 5 ppm Cu compared to those received 200 ppm Cu as TBCC [65]. In the current study, high Cu supplementation in MMHAC form (30 ppm) increased the thigh and drumstick processing weights, while inorganic Cu (TBCC, 125 ppm) did not affect carcass weight. Thus, feeding MMHACs at 30 ppm would be more beneficial in improving carcass processing weight in birds compared to inorganic Cu sources. Previous work has demonstrated that chelated trace minerals were able to increase carcass integrity and reduce wooden breast likely through improving the antioxidant status and tissue healing process [40]. No differences were noted in carcass quality characteristics among dietary treatments in this study, possibly due to the lack of meat quality defects reflected by no breast blisters and skin scratches leaving little room for improvement.

It has been reported that high dietary Cu levels may induce gizzard erosion and that gizzard erosion severity increases with the dietary Cu level [66]. It is possible that high inorganic Cu levels reduce gizzard pH thereby affecting the integrity of the gizzard lining [67]. Furthermore, Swirski et al. [30] indicated that gizzard erosion reduces birds’ productivity by impairing feed efficiency and increasing morbidity and mortality rates. In the current study, although the gizzard erosion score at day 42 and the liveability rate and FCR from days 0 to 42 of birds fed the T125 diet were numerically poorer than the other treatments, significant differences were not detected for these variables.

Trace minerals play crucial roles in bone development [68]. The results of this study demonstrated that reduced levels of MMHACs were sufficient to maintain bone quality compared to the control diet with high inorganic mineral levels. Similar results were reported by El-Husseiny et al. [69], who observed a similar tibia weight, length, and diameter in birds that received 50% of organic Cu, Zn, and Mn compared to birds that received 100% inorganic Zn, Mn, and Cu at day 35. Likewise, Ghasemi et al. [11] indicated that the tibia length, weight, and ash content were not different between birds offered inorganic trace minerals at commercially recommended levels and those offered chelated trace minerals, which matched 25, 50, and 100% of inorganic trace mineral diet at day 42.

The determination of trace mineral digestibility using indigestible markers such as titanium dioxide is generally difficult due to the intricate nature of endogenous mineral excretion [68]. Thus, similarly to this study, negative ileal digestibility values could be seen for Cu, Zn, and Mn when indigestible markers were used in other poultry nutrition studies [68,70]. Nevertheless, due to the safety, post-recovery rate, and reproducibility of measurements of titanium dioxide compared to the other digestibility markers, titanium dioxide has been considered valid and the most suitable marker to determine nutrient digestibility in the majority of animal feeding trials [71,72]. It is known that endogenous trace mineral flow is influenced by the bird age, dietary mineral level, and intestinal segments [68]. The negative Mn and Zn ileal digestibility noted in this study suggests that the endogenous flow of Mn and Zn may be higher than the absorption of these minerals in the ileum on day 21. Furthermore, previous studies have suggested that Mn may be mainly digested in the duodenum and that its absorption rate is very low depending on levels of other trace minerals, not its dietary level [68]. Similarly, as the duodenum is the first intestinal segment where elements that come from the diet, including Zn, are apparently absorbed, leaving little Zn left to enter and be digested in the ileum [68]. However, the increased Cu digestibility in birds offered the M30 diet in the current study may stem from the higher digestible and bioavailable properties of organic trace minerals resulting from fewer antagonisms and interactions with other dietary components such as phytate, Ca, and/or P during the nutrient digestion compared to the inorganic counterparts [7,73]. Similar results were observed in previous studies [11,68]. For example, M’Sadeq et al. [68] reported that birds fed diets with added organic yeast proteinate trace mineral premix had higher Cu digestibility in the jejunum but similar Mn and Zn digestibility in the ileum and jejunum compared to those fed inorganic trace mineral sources on day 25 [68]. Likewise, Ghasemi et al. [11] reported higher apparent ileal Cu digestibility in birds offered chelated trace minerals at 25% of the inorganic trace mineral levels compared to those offered 100% inorganic trace minerals on day 35. The findings of this study also showed that birds fed the M30 diet had higher apparent ileal Cu digestibility compared to those fed the M10 diet. This finding was consistent with those observed by M’Sadeq et al. [68], who reported higher apparent ileal Cu digestibility in birds fed the higher level of organic yeast proteinate trace mineral premix (500 g/ton) compared to those fed the lower level of this mineral source (275 g/ton) on day 25. Likewise, the findings of this study are supported by Kim et al. (2011), who observed higher Cu accumulation in the liver in broilers fed Cu-methionine chelate at 100 ppm compared to those fed the lower level of Cu-methionine chelate (50 ppm) suggesting the higher Cu absorption in the respective treatment. This may be associated with the beneficial effects of chelated Cu supplementation at higher levels on increasing the number of beneficial bacteria such as *Lactobacillus* and decreasing the number of pathogenic bacteria such as *Escherichia coli* and *Enterobacteriaceae* in the gut leading to reduced inflammation and increased nutrient absorption [33,74]. Such a mechanism is similar to that of probiotic supplementation in broiler production [75].

## 5. Conclusions

Although significant differences were not obtained, the findings of the current study show that the supplementation of MMHACs to broiler diets at 30 ppm possibly increased BWG and FI compared to the ITM diet. Furthermore, feeding the M30 diet significantly increased thigh and drumstick processing weight and Cu digestibility compared to the ITM diet while maintaining the bone health of broilers. Birds offered the M30 diet could achieve a similar level of growth performance compared to those offered the T125 diet, although Cu, Zn, and Mn levels in this diet were reduced. It was also found that feeding the T125 diet was more beneficial during the grower phase in improving feed efficiency; however, during the finisher phase, feeding this diet did not generate any beneficial effects. Hence, the dietary supplementation of MMHACs at 30 ppm was the most suitable nutritional strategy to improve the weight gain and carcass processing weight in broiler production in the present study.

## Figures and Tables

**Table 1:** Description of dietary treatments.

Treatment Number	Treatment Code	Trace Mineral Level and Source
1	ITM	Inorganic trace mineral 110 ppm Zn as ZnSO_4_, 16 ppm Cu as CuSO_4_, and 120 ppm Mn as MnO per Ross 308 nutritional recommendations [36].
2	M10	Amounts of 40 ppm Zn, 10 ppm Cu, and 40 ppm Mn as chelate (MINTREX^®^ *).
3	T125	Inorganic trace mineral 110 ppm Zn as ZnSO_4_ and 120 ppm Mn as MnO per Ross 308 guidelines, with 125 ppm Cu as tribasic copper chloride (TBCC).
4	M30	Amounts of 40 ppm Zn, 30 ppm Cu, and 40 ppm Mn as chelate (MINTREX^®^).

* MINTREX^®^ Zn, Cu, and Mn trace minerals from Novus International, Inc. (Chesterfield, MO, USA) with Zn, Cu, and Mn bound by methionine hydroxyl analogues in a 2:1 chelated molecule.

**Table 2:** Diet composition and calculated nutrient values of the basal/control diets (as-fed basis).

Ingredients %	Starter (Days 0–10)	Grower (Days 10–21)	Finisher (Days 21–42)
Wheat	38.89	34.37	38.94
Sorghum	20.00	30.00	30.00
Soybean meal	34.33	29.09	25.65
Canola oil	2.84	2.92	2.83
Limestone	1.14	0.86	0.79
Dicalcium phosphate	1.13	0.75	0.45
Xylanase (Econase XT 5P)	0.002	0.002	0.002
Phytase (Quantum Blue 5G)	0.01	0.01	0.01
Salt	0.20	0.17	0.19
Sodium bicarbonate	0.23	0.17	0.16
Titanium dioxide	0.00	0.50	0.00
Vitamin–mineral premix ^1^	0.10	0.10	0.10
Choline Cl 60%	0.048	0.054	0.041
L-lysine HCl	0.362	0.341	0.309
D,L-methionine	0.438	0.395	0.351
L-threonine	0.176	0.149	0.118
L-arginine	0.050	0.067	0.056
L-valine	0.056	0.044	0.023
Calculated nutrient % (otherwise as stated)			
Dry matter	91.0	90.9	90.9
AMEn ^2^, kcal/kg	2975	3050	3100
Crude protein	22.9	20.9	19.7
Crude fat	4.76	5.05	5.02
Crude fibre	3.20	3.42	3.39
Ash content	4.68	4.47	3.50
Dig. ^3^ lysine	1.320	1.180	1.080
Dig. methionine	0.708	0.647	0.592
Dig. methionine + cysteine	1.000	0.920	0.860
Dig. threonine	0.880	0.790	0.720
Calcium	0.950	0.750	0.650
Available phosphorus	0.500	0.420	0.360
Sodium	0.210	0.180	0.180
Potassium	1.013	0.918	0.863
Chloride	0.250	0.230	0.230
Choline, mg/kg	1700	1600	1500
Linoleic acid	1.686	1.803	1.813
Dietary electrolyte balance, mEq/kg	302	269	253

^1^ Vitamin–mineral premixes used in the experimental diets were prepared and provided by an experienced manufacturer (Rabar Pty Ltd., Beaudesert, QLD, Australia). The vitamin–mineral premix in the basal/control diet was replaced with the respective vitamin–mineral premix for each treatment to generate M10, T125, and M30 diets. Levels of supplemental D,L-methionine were reduced in the M10 and M30 diets to generate similar methionine levels across the treatments; ^2^ AMEn: apparent metabolizable energy corrected to zero N retention; ^3^ Dig: standard ileal digestible amino acid coefficients as determined by Near-Infra Red spectroscopy (Foss NIR 6500, Hillerød, Denmark) standardised with Adisseo calibration.

**Table 3:** Analysed nutrient values of experimental diets (as-fed basis).

Nutrients	Starter (Days 0–10)				Grower (Days 10–21)				Finisher (Days 21–42)			
	ITM	M10	T125	M30	ITM	M10	T125	M30	ITM	M10	T125	M30
DM, %	89.3	88.6	89.5	89.2	89.5	89.7	90.3	89.0	89.7	89.6	89.6	89.7
GE, kcal/kg	4039	3997	4025	4021	4038	4046	4079	4000	4074	4082	4083	4084
CP, %	22.0	22.0	22.8	22.0	20.8	21.0	21.5	21.2	19.5	19.3	19.5	19.8
Cu, µg/g	19.8	20.2	123.8	55.1	21.7	20.5	135.5	45.8	18.2	20.9	137.2	44.8
Zn, µg/g	115.2	71.9	117.6	72.2	124.2	68.1	119.6	60.9	123.1	57.9	122.9	58.9
Mn, µg/g	166	137	167	139	203	121	192	117	172	111	171	105
P, %	0.66	0.67	0.66	0.66	0.60	0.59	0.59	0.57	0.51	0.52	0.51	0.50
Ca, %	0.74	0.84	0.85	0.81	0.64	0.68	0.63	0.60	0.57	0.58	0.52	0.52
Mg, %	0.19	0.19	0.18	0.18	0.18	0.18	0.18	0.18	0.17	0.17	0.17	0.16
Na, %	0.13	0.15	0.15	0.14	0.12	0.13	0.12	0.12	0.13	0.14	0.12	0.12
K, %	1.13	1.09	1.07	1.08	1.00	0.99	1.00	0.99	0.92	0.92	0.91	0.90
S, %	0.32	0.33	0.34	0.32	0.31	0.30	0.31	0.29	0.30	0.29	0.28	0.28
Al, µg/g	68.5	71.8	71.6	65.6	52.7	53.2	53.6	49.6	51.9	47.8	43.9	44.5
Fe, µg/g	99.5	114.3	108.9	102.0	100.3	98.1	99.6	89.8	89.2	90.4	86.7	88.1
B, µg/g	17.0	16.5	15.9	15.9	15.5	15.4	15.6	15.4	13.8	13.7	12.5	11.9
Cr, µg/g	0.38	0.46	0.40	0.39	1.24	1.15	1.23	1.06	0.57	0.58	0.34	0.27
Co, µg/g	2.24	1.07	0.90	0.67	0.91	0.69	0.87	0.61	0.78	0.65	0.65	0.50

ITM: inorganic trace mineral ZnSO_4_ 110 ppm, CuSO_4_ 16 ppm, MnO 120 ppm; M10: mineral methionine hydroxyl analogue chelates Zn 40 ppm, Cu 10 ppm, Mn 40 ppm; T125: inorganic trace mineral ZnSO_4_ 110 ppm, tribasic copper chloride 125 ppm, MnO 120 ppm; M30: mineral methionine hydroxyl analogue chelates Zn 40 ppm, Cu 30 ppm, Mn 40 ppm; DM: dry matter; GE: gross energy; CP: crude protein.

**Table 4:** Growth performance of broilers fed inorganic and methionine hydroxyl analogue chelated zinc, copper, and manganese from days 0 to 42.

Feeding Phase	Variable	ITM	M10	T125	M30	SEM	*p*-Value
Starter (days 0–10)	Weight gain (g/bird)	288	309	292	301	7.93	0.308
	Feed intake (g/bird)	294	316	297	309	8.71	0.312
	FCR	1.020	1.022	1.016	1.028	0.006	0.665
	Liveability rate (%)	96.2	98.1	98.1	98.1	1.82	0.675
Grower (days 10–21)	Weight gain (g/bird)	838	848	869	878	17.10	0.382
	Feed intake (g/bird)	1042	1081	1041	1085	14.93	0.086
	FCR	1.244 ^ab^	1.278 ^b^	1.201 ^a^	1.237 ^ab^	0.014	0.021
	Liveability rate (%)	99.0	99.0	95.8	99.0	1.18	0.188
Finisher (days 21–42)	Weight gain (g/bird)	2312	2427	2443	2442	44.69	0.155
	Feed intake (g/bird)	4438	4557	4768	4682	82.11	0.057
	FCR	1.924	1.884	1.952	1.920	0.041	0.725
	Liveability rate (%)	97.2	97.2	91.1	94.4	2.38	0.085
Overall (days 0–42)	Weight gain (g/bird)	3441	3583	3612	3625	49.79	0.063
	Feed intake (g/bird)	5774	5954	6106	6077	95.87	0.052
	FCR	1.679	1.664	1.690	1.677	0.023	0.888
	Liveability rate (%)	93.3	95.2	88.5	93.3	2.26	0.244
Overall liveability and European productivity index (days 0–42)		462	494	455	487	16.63	0.308
Coefficient of variation in mean individual body weight at day 42		8.12	7.13	6.47	5.77	1.03	0.499

ITM: inorganic trace mineral ZnSO_4_ 110 ppm, CuSO_4_ 16 ppm, MnO 120 ppm; M10: mineral methionine hydroxyl analogue chelates Zn 40 ppm, Cu 10 ppm, Mn 40 ppm; T125: inorganic trace mineral ZnSO_4_ 110 ppm, tribasic copper chloride 125 ppm, MnO 120 ppm; M30: mineral methionine hydroxyl analogue chelates Zn 40 ppm, Cu 30 ppm, Mn 40 ppm; FCR: feed conversion ratio. ^a,b^ Differing superscripts within a row indicate significant differences between means. *p*-values ≤ 0.05 were considered significant.

**Table 5:** Water intake of broilers fed inorganic and methionine hydroxyl analogue chelated zinc, copper, and manganese from days 0 to 42.

Feeding Phase	Variable	ITM	M10	T125	M30	SEM	*p*-Value
Starter (days 0–10)	Water intake (L/bird)	0.73	0.73	0.76	0.73	0.03	0.872
	Water intake (L/kg BW)	2.52	2.38	2.52	2.42	0.09	0.625
	Water/feed intake	2.47	2.33	2.47	2.35	0.08	0.501
Grower (days 10–21)	Water intake (L/bird)	1.96	2.09	1.96	2.04	0.04	0.182
	Water intake (L/kg BW)	2.32	2.45	2.25	2.34	0.05	0.057
	Water/feed intake	1.87	1.93	1.88	1.89	0.02	0.529
Finisher (days 21–42)	Water intake (L/bird)	6.65 ^a^	7.03 ^ab^	6.70 ^ab^	7.06 ^b^	0.11	0.020
	Water intake (L/kg BW)	2.86	2.90	2.79	2.87	0.04	0.464
	Water/feed intake	1.49	1.55	1.45	1.52	0.03	0.117
Overall (days 0–42)	Water intake (L/bird)	9.32 ^a^	10.07 ^b^	9.39 ^a^	9.83 ^ab^	0.13	0.002
	Water intake (L/kg BW)	2.71 ^ab^	2.78 ^b^	2.60 ^a^	2.70 ^ab^	0.03	0.010
	Water/feed intake	1.59 ^ab^	1.68 ^b^	1.57 ^a^	1.63 ^ab^	0.02	0.046

ITM: inorganic trace mineral ZnSO_4_ 110 ppm, CuSO_4_ 16 ppm, MnO 120 ppm; M10: mineral methionine hydroxyl analogue chelates Zn 40 ppm, Cu 10 ppm, Mn 40 ppm; T125: inorganic trace mineral ZnSO_4_ 110 ppm, tribasic copper chloride 125 ppm, MnO 120 ppm; M30: mineral methionine hydroxyl analogue chelates Zn 40 ppm, Cu 30 ppm, Mn 40 ppm; BW: Body weight. ^a,b^ Differing superscripts within a row indicate significant differences between means. *p*-values ≤ 0.05 were considered significant.

**Table 6:** Carcass processing weight (g/bird) and woody breast and breast white striping scores of broilers fed inorganic and methionine hydroxyl analogue chelated zinc, copper, and manganese dietary treatments at day 42.

Variable	ITM	M10	T125	M30	SEM	*p*-Value
Breast	684	716	719	713	14.86	0.415
Thigh	397	413	412	425	7.29	0.078
Drumstick	320	335	332	340	4.68	0.058
Thigh and drumstick	717 ^a^	748 ^ab^	745 ^ab^	765 ^b^	11.32	0.050
Fat pad	32.3	33.1	33.4	35.5	1.59	0.546
Liver	68.0	67.4	66.0	71.1	2.41	0.528
Heart	18.8	18.6	19.2	19.2	0.68	0.898
Kidney	9.49	10.2	10.6	10.6	0.51	0.413
Woody breast score	0.792	0.583	0.917	0.583	0.148	0.337
Breast white striping score	1.917	1.583	1.750	1.750	0.223	0.788

ITM: inorganic trace mineral ZnSO_4_ 110 ppm, CuSO_4_ 16 ppm, MnO 120 ppm; M10: mineral methionine hydroxyl analogue chelates Zn 40 ppm, Cu 10 ppm, Mn 40 ppm; T125: inorganic trace mineral ZnSO_4_ 110 ppm, tribasic copper chloride 125 ppm, MnO 120 ppm; M30: mineral methionine hydroxyl analogue chelates Zn 40 ppm, Cu 30 ppm, Mn 40 ppm. ^a,b^ Differing superscripts within a row indicate significant differences between means. *p*-values ≤ 0.05 were considered significant.

**Table 7:** Apparent ileal nutrient digestibility of broilers fed inorganic and methionine hydroxyl analogue chelated zinc, copper, and manganese at day 21.

Nutrient Digestibility Coefficient	ITM	M10	T125	M30	SEM	*p*-Value
Cu	0.046 ^a^	−0.001 ^a^	0.207 ^ab^	0.250 ^b^	0.053	0.006
Zn	−0.040	−0.062	−0.039	−0.014	0.045	0.902
Mn	−0.001	−0.114	−0.033	−0.020	0.047	0.371
P	0.637	0.669	0.676	0.655	0.017	0.428
Ca	0.567	0.559	0.575	0.532	0.024	0.599
Nitrogen (protein)	0.846	0.854	0.858	0.849	0.005	0.314
Energy	0.762	0.775	0.768	0.761	0.006	0.293

ITM: inorganic trace mineral ZnSO_4_ 110 ppm, CuSO_4_ 16 ppm, MnO 120 ppm; M10: mineral methionine hydroxyl analogue chelates Zn 40 ppm, Cu 10 ppm, Mn 40 ppm; T125: inorganic trace mineral ZnSO_4_ 110 ppm, tribasic copper chloride 125 ppm, MnO 120 ppm; M30: mineral methionine hydroxyl analogue chelates Zn 40 ppm, Cu 30 ppm, Mn 40 ppm. ^a,b^ Differing superscripts within a row indicate significant differences between means. *p*-values ≤ 0.05 were considered significant.

## Data Availability

The data that support this study will be shared upon reasonable request with the corresponding author.

## Appendix A

**Table A1:** Tibia morphological parameters and breaking strength of broilers fed inorganic and methionine hydroxyl analogue chelated zinc, copper, and manganese at day 42.

Variable	ITM	M10	T125	M30	SEM	*p*-Value
Air-dry weight (g)	10.3	10.3	10.4	10.3	0.20	0.984
Oven-dry weight (g)	9.09	9.05	9.10	8.98	0.17	0.961
Ash content (%)	40.6	41.1	40.9	40.9	0.42	0.887
Length (mm)	104.5	104.7	104.8	104.8	0.70	0.993
Diameter (mm)	10.9	10.8	11.0	10.7	0.16	0.567
Breaking strength (N)	463	485	498	457	19.39	0.417

ITM: inorganic trace mineral ZnSO_4_ 110 ppm, CuSO_4_ 16 ppm, MnO 120 ppm; M10: mineral methionine hydroxyl analogue chelates Zn 40 ppm, Cu 10 ppm, Mn 40 ppm; T125: inorganic trace mineral ZnSO_4_ 110 ppm, tribasic copper chloride 125 ppm, MnO 120 ppm; M30: mineral methionine hydroxyl analogue chelates Zn 40 ppm, Cu 30 ppm, Mn 40 ppm. Tibia ash content was calculated as percentage of oven-dry tibia. Tibia length, diameter, and breaking strength were measured on air-dry tibias.
